# Hypereosinophilic syndrome with massive liver infarction: A case report

**DOI:** 10.1097/MD.0000000000035903

**Published:** 2023-11-17

**Authors:** Shan-Yu Lu, Yi-Fan Hua, Li Guo

**Affiliations:** a Department of Radiology, Second Affiliated Hospital of Kunming Medical University, Kunming, Yunnan, P.R. China.

**Keywords:** glucocorticoid pulse therapy, hypereosinophilic syndrome, interventional therapy, liver infarction

## Abstract

**Rationale:**

Liver infarction caused only by hepatic artery occlusion is rare. Elevated levels of eosinophils in the blood and tissue can have devastating consequences.

**Patient concerns:**

Male, 21 years old, presented with persistent abdominal distension and discomfort for more than ten days without an apparent cause. Laboratory findings showed an eosinophil percentage of 32.5% (normal range 0.5%–5%). Computed tomographic angiography of the hepatic artery and its branches did not show any enhancement, only the common hepatic artery was visible.

**Diagnosis:**

The patient in this case had a peripheral blood eosinophil count of ≥1.5 × 109/L in multiple examinations over 6 months, and eosinophilic leukemia and secondary causes such as parasitic infections, allergic diseases, or tumors were ruled out, confirming the diagnosis of hypereosinophilic syndrome (HES).

**Interventions:**

The patients were treated with interventional therapy, glucocorticoid pulse therapy and anti-infection therapy.

**Outcomes:**

After interventional therapy, glucocorticoid pulse therapy, and anti-infection treatment, the patient was reexamined 2 months later. The CT scan showed that the range of the original infarction in the liver had shrunk compared to before, and the remaining liver had enlarged with good compensation; Laboratory tests improved compared with baseline: eosinophil percentage of 0.1%.

**Lessons:**

This article discusses a rare case of hepatic artery occlusion and liver infarction in a young male patient with HES. The cause of hepatic artery embolism and hepatic infarction may be related to the abnormal increase in eosinophils, which can lead to hypercoagulation and thrombus formation. The article emphasizes the importance of timely diagnosis and treatment of HES to prevent life-threatening thrombotic events and describes the successful management of the patient condition through anticoagulation, anti-infection, liver protection, and glucocorticoid therapy.

## 1. Introduction

Elevated levels of eosinophils in the blood and tissue can have devastating consequences, as evidenced by the case of a young male with hypereosinophilic syndrome (HES) who developed hepatic artery occlusion and liver infarction. While hepatic infarction caused solely by hepatic artery occlusion is rare, the extensive occlusion of the hepatic artery and its branches seen in this case, coupled with the failure to establish timely collateral circulation, may have contributed to the severe outcome. Although glucocorticoid therapy was administered after the occurrence of hepatic infarction, it is likely that the hypercoagulable state caused by the continuous abnormal increase of eosinophils in peripheral blood and liver tissue was the main mechanism behind the large area of hepatic infarction. Clinicians should be reminded that HES can cause thrombotic symptoms and prompt intervention measures should be taken to prevent life-threatening complications.

## 2. Case

**Figure 1.**
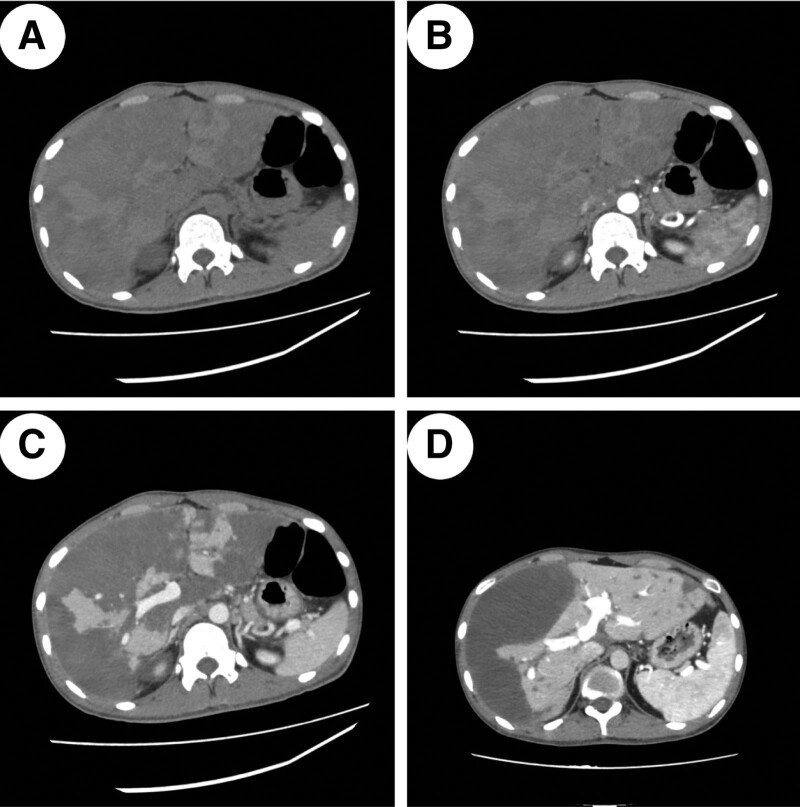
Hypereosinophilic Syndrome with extensive hepatic infarction. (A–C) Sagittal CT images showing plain scan, arterial phase enhanced scan, and portal venous phase enhanced scan, respectively. (D) Follow-up portal venous phase enhanced CT scan 2 months later.

Male, 21 years old, presented with persistent abdominal distension and discomfort for more than ten days without an apparent cause. Laboratory findings showed a white blood cell count of 11.65 × 10^9^/L (normal range 15–46 × 10^9^/L), an eosinophil percentage of 32.5% (normal range 0.5%–5%), alanine aminotransferase of 7129 U/L (normal range 0–50 U/L), aspartate aminotransferase of 10211 U/L (normal range 0–50 U/L), quantitative d-dimer >45 ug/mL (normal range 0–0.55 ug/mL), and serum total IgE level of 170.0 IU/mL (normal range <165 IU/mL). Nonenhanced computed tomography and contrast-enhanced CT showed multiple wedge-shaped and irregular low-density shadows in the liver, with their bases facing the edge of the liver, tips pointing to the liver portal, clear borders, and no enhancement (Fig. 1A–C). Computed tomographic angiography of the hepatic artery and its branches did not show any enhancement, only the common hepatic artery was visible, and the portal vein was unobstructed without any filling defects. The imaging diagnosis was liver infarction due to hepatic artery occlusion. After interventional therapy, glucocorticoid pulse therapy, and anti-infection treatment, the patient was reexamined 2 months later. The CT scan showed that the range of the original infarction in the liver had shrunk compared to before, and the remaining liver had enlarged with good compensation (Fig. 1D); All laboratory tests improved compared with baseline: white blood cell count of 6.19 × 10^9^/L, eosinophil percentage of 0.1%, alanine aminotransferase of 74 U/L, aspartate aminotransferase of 46 U/L, quantitative d-dimer of 0.42 ug/mL, and serum total IgE level of 127.0 IU/mL.

## 3. Discussion

HES is a group of diseases characterized by a persistent increase of eosinophils in blood and bone marrow, and eosinophil infiltration in tissues, with unknown etiology and multiple system involvement. The diagnostic criteria for HES include: peripheral blood eosinophil count ≥1.5 × 10^9^/L at least twice; exclusion of parasitic, allergic, or other diseases that may cause eosinophilia; symptoms and signs of systemic involvement.^[1]^ The patient in this case had a peripheral blood eosinophil count of ≥1.5 × 10^9^/L in multiple examinations over 6 months, and eosinophilic leukemia and secondary causes such as parasitic infections, allergic diseases, or tumors were ruled out, confirming the diagnosis of HES.

During the active phase of the disease, the patient in this case also developed hepatic artery occlusion and liver infarction. Was this a coincidence or was there a causal relationship between the increased eosinophils and the liver infarction? The liver has a dual blood supply and liver infarction is rare clinically. Hepatic artery occlusion can be caused by thrombosis, embolism, blunt liver injury, and vascular damage during surgery (such as arterial chemoembolization and liver transplantation), but this patient was a young male with no common high-risk factors for thrombosis formation except for HES. A review of domestic and foreign literature found that HES complicated with thrombotic events is rare but has been reported in several cases, and it is considered that eosinophils may play an important role in thrombus formation.^[2–4]^ Therefore, we speculate that the occurrence of hepatic artery embolism and hepatic infarction in this patient may be related to the abnormal increase in eosinophils. Of course, other causes of hepatic artery embolism cannot be completely ruled out. However, from the course of the disease, the elevation of eosinophils overlaps with the time of hepatic artery embolism, and the causal relationship is more likely.

In this case, we observed the occlusion of the hepatic intrinsic artery and its branches, accompanied by a large area of liver tissue infarction, which is extremely rare in hepatic infarction caused by hepatic artery occlusion. Since the blood supply to the liver mainly comes from the portal vein (about 75%), and the remaining part comes from the hepatic artery (about 25%), hepatic infarction caused only by hepatic artery occlusion is rare, and the range and degree are relatively mild. We speculate that the occurrence of hepatic artery occlusion and a large area of hepatic infarction may be related to the extensive occlusion of the hepatic artery and its branches, the failure to establish timely collateral circulation in the posterior branches, the obstruction of hepatic tissue microcirculation, and the ineffective compensation of the portal vein blood supply for the decrease in the hepatic artery. These may all be related to the thrombotic state of HES, and its possible mechanisms include the toxic effect of eosinophils leading to hypercoagulation; the induction of endothelial cells by eosinophils to secrete various inflammatory mediators, resulting in local thrombosis formation; IgE can induce platelet activation, and HES patients often have elevated blood IgE levels; the application of the glucocorticoid system leads to a hypercoagulable state. The systemic application of glucocorticoids in this patient was used after the occurrence of hepatic infarction, therefore, the cause of hypercoagulability due to glucocorticoid use can be preliminarily excluded.^[4,5]^ Considering the continuous abnormal increase in eosinophils in peripheral blood and liver tissue and the resulting hypercoagulable state, we believe that this is the main mechanism for the occurrence of a large area of hepatic infarction in this patient, but it is unfortunate that liver tissue biopsy was not performed to confirm this. We also hope that similar cases in the future can confirm this hypothesis.

## 4. Conclusion

Large-area hepatic infarction can cause coma, ascites, jaundice, liver and kidney failure, and the prognosis is extremely poor. After the diagnosis of hepatic artery occlusion and large area hepatic infarction caused by HES, immediately reinforcing the primary treatment, and anticoagulation, anti-infection, liver protection, and glucocorticoid therapy were given to prevent further aggravation of liver damage and the occurrence of multiple organ dysfunction. After treatment, the patient condition improved significantly: blood and biochemical indicators decreased, the infarcted liver tissue shrank, and the compensated liver tissue proliferated. These should be related to the timely diagnosis (imaging is more characteristic), reasonable treatment, and the patient youth and strong liver function compensatory ability. Reporting this case aims to remind clinical and imaging doctors that HES itself can cause thrombotic symptoms. Failure to diagnose and treat it in a timely manner can be life-threatening. When various organs experience infarction, it may be an early manifestation of thrombotic diseases. Timely intervention measures should be taken into consideration.

## Author contributions

**Conceptualization:** Shan-Yu Lu, Yi-Fan Hua, Li Guo.

**Supervision:** Li Guo.

**Validation:** Shan-Yu Lu, Yi-Fan Hua, Li Guo.

**Visualization:** Shan-Yu Lu, Yi-Fan Hua.

**Writing – original draft:** Shan-Yu Lu, Yi-Fan Hua.

**Writing – review & editing:** Shan-Yu Lu, Yi-Fan Hua, Li Guo.
